# Florfenicol Binding to Molecularly Imprinted Polymer Nanoparticles in Model and Real Samples

**DOI:** 10.3390/nano10020306

**Published:** 2020-02-11

**Authors:** Nelson Caro, Tamara Bruna, Antonio Guerreiro, Paola Alvarez-Tejos, Virginia Garretón, Sergey Piletsky, Jorge González-Casanova, Diana Rojas-Gómez, Nicole Ehrenfeld

**Affiliations:** 1 Centro de Investigación Austral Biotech, Universidad Santo Tomas, Avenida Ejercito 146, Santiago 7591538, Chile; ncaro@australbiotech.cl (N.C.);; 2 Departament of Chemistry, University of Leincester, Leicester LE1 7RH, UK; 3 Instituto de Ciencias Biomédicas, Facultad de Ciencias de la Salud, Universidad Autónoma de Chile, Santiago 7591538, Chile; 4 Escuela de Nutrición y Dietética, Facultad de Medicina, Universidad Andres Bello, Santiago 7591538, Chile

**Keywords:** nanoparticles, polymer, florfenicol

## Abstract

A simple and straightforward technique for coating microplate wells with molecularly imprinted polymer nanoparticles (nanoMIPs) to develop assays similar to the enzyme-linked immunosorbent (ELISA) assay to determine and quantify florfenicol (FF) in real food samples such as liquid milk and salmon muscle is presented here. The nanoMIPs were synthesized by a solid-phase approach with an immobilized FF (template) and characterized using dynamic light scattering, a SPR-2 biosensor system and transmission electron microscopy. Immobilization of nanoMIPs was conducted by preparing a homogenous solution of FF-nanoMIPs in water mixed with polyvinyl alcohol (PVA) 0.2% (*w/v*) in each well of a microplate. The detection of florfenicol was achieved in competitive binding experiments with a horseradish peroxidase−florfenicol (FF–HRP) conjugate. The assay made it possible to measure FF in buffer and in real samples (liquid milk and salmon muscle) within the range of 60−80 and 90–100 ng/mL, respectively. The immobilized nanoMIPs were stored for six weeks at room temperature and at 5 °C. The results indicate good signal recovery for all FF concentrations in spiked milk samples, without any detrimental effects to their binding properties. The high affinity of nanoMIPs and the lack of a requirement for cold chain logistics make them an attractive alternative to traditional antibodies used in ELISA.

## 1. Introduction

Bacterial resistance to antimicrobials has become an important public interest and a scientific problem since the last decade. Although there is no consensus on the degree of influence of the use of antimicrobials in animals on the occurrence and spread of antimicrobial resistant bacteria in humans, experimental evidence as well as epidemiological and molecular studies indicate a relationship between the use of antimicrobials and the appearance of resistant bacterial strains in animals and their spread to humans, especially through the food chain. There are risks related to the appearance of bacterial resistance, as the accumulation of antimicrobial residues in animal products can damage human health. Currently, antimicrobial chemotherapy in veterinary medicine is the main therapeutic tool used against pathogenic microorganisms [1]. Antibiotics are used prophylactically and metaphylactically [2,3,4]. In aquaculture, and especially in salmon farming, antibiotics are orally administered through feedstuffs, with florfenicol (FF) being the main antibiotic used (52%), followed by oxytetracycline, flumequine and oxolinic acid (44%, 1% and 1%, respectively) [5,6]. FF, a fluorinated derivative of thiamphenicol, is a new generation of fenicol drugs [7]. Apart from in the aquaculture industry, FF is widely used as chloramphenicol alternative to prevent and treat bacterial diseases in pigs [8], bovine [9], poultry [10] and animal edible tissues [11]. Several countries adopted maximum permissible residue limits (MRLs) of antibiotics in foods of animal origin, such as meat, milk and eggs [12]. In Chile, the maximum permissible residue limit of FF in muscle and skin of salmon is 1000 µg/kg [13]. At present, no MRL has been set for FF in milk. It is however of vital importance to develop a reliable method for FF analysis in milk products to avoid potential harmful effects to consumers [14]. Therefore, there is a demand for relevant sensitive, selective, fast and inexpensive analytical protocols from legislation, health authorities and companies operating in the food market [15]. Various analytical techniques have been reported for the analysis of FF, including high performance liquid chromatography, gas chromatography, liquid chromatography—Tandem mass spectrometry [16], gas chromatography—Mass spectrometry [17], and enzyme-linked immunosorbent assay (ELISA) [18]. The manufacture of molecular imprinting polymers (MIP) can be very valuable for those selective and sensitive methods that are needed. Molecular impression is a technique for producing in a polymer matrix selective binding sites that recognize a particular molecule. These polymers are very stable, as they are not degraded by enzymatic action, heat, pH, pressure or in organic solvents. Other advantages of these materials are the low cost of reagents necessary for their preparation and easy manufacturing, so that they can be produced in large quantities.

Molecularly imprinted polymers (MIPs) are a new class of affinity receptors that contain selective binding sites for the corresponding target analytes [19]. The shape, size and chemical functionality of MIPs binding sites are complementary to the target analyte that interacts with polymers through a hydrogen bond, ion-exchange, electrostatic interaction, etc. MIPs exhibit high selectivity, sensitivity, stability and binding capacity [20]. The notable advantages of MIPs which have attracted attention from researchers and engineers are the low cost of production and their potential for use in separation and detection applications [21,22]. Other advantages are their predetermined recognition ability and resistance to a wide range of pH, solvents, and temperature [19,23]. Several applications have been described for bulk MIPs and MIP nanoparticles (nanoMIPs), which include analysis of analytes in environmental and biological samples, solid-phase extraction [24], chromatography [25], enantiomeric separations [26] and sensors applications [27]. In addition, several examples of the application of MIPs in microplate-based assays have been described [28]. The main advantage of nanoMIPs in diagnostics is the possibility of direct replacement of antibodies in standard ELISA like assays with minimal modification of the immobilization and assay protocol [29]. This work describes the development of nanoMIPs for FF and analysis of their binding in real samples such as salmon and milk. The system based on nanoMIPs can potentially allow replacing antibodies in ELISA-type assay. The following aspects of this system were investigated: (i) the physicochemical characteristics of nanoparticles, their affinity (K_D_) and cross-reactivity; and (ii) performance in binding assay where free FF from foodstuff competes for binding to nanoMIPs with HRP-FF conjugate. The decrease in HRP-generated signal was linked to the amount of free FF available in food samples.

## 2. Materials and Methods

### 2.1. MIPs and NIPs Synthesis

Florfenicol (FF) was immobilized onto the surface of glass beads (90 µm diameter) containing epoxy groups. For this, FF was dissolved in acetone followed by the addition of carbonate buffer (pH 12.0) and then the beads, at final concentration of 0.5 mg/mL, and incubated for 12 h. The composition of the polymerization mixture of florfenicol-nanoMIPs was adapted from a previous work [30,31]. For the synthesis of NIPs (non-imprinted polymer), FF was replaced with melamine. For the preparation of blank polymers in the solid-phase synthesis, there is a need to have unrelated anchored molecule to be present during the synthesis. If the solid support has no template attached, then no polymer can be recovered. For the synthesis, 35 g of derivatized beads were placed in contact with polymerization mixture which contained: methacrylic acid (1.4 g), ethylene glycol dimethacrylate (1.6 g) and trimethylolpropane trimethacrylate (1.6 g) as cross-linkers; benzyl dithiocarbamate (initiator, 0.3 g), pentaerythrioltetrakis(3-mercaptopropionate) as a chain transfer agent (0.07 g); and acetonitrile (ACN) (5.2 g). The mixture was irradiated under UV for 2 min. The beads containing nanoparticles attached to the template were then washed with four bead volumes of acetonitrile at 22 °C, placed in acetonitrile (10 mL, with 9 mg/mL polyethylene glycol (PEG) acrylate 1100), and irradiated for 30 s (Philips HB/171/A with 4 × 15 W lamps). This was done to create an outer shell which facilitates the physical immobilization of the nanoparticles onto the microplates. After the second irradiation step, glass beads containing bound nanoparticles were placed in a solid phase extraction (SPE) tube with polyethylene frit and washed with 8–9 bed volumes of acetonitrile at 22 °C to remove unreacted monomers, until no material could be detected spectrophotometrically in the washing solution. After the washing step, the beads were warmed up to 70 °C, and washed with five bed volumes of acetonitrile at the same temperature, to elute affinity nanoparticles. This procedure was repeated five times, and the resultant nanoparticles solutions in ACN then was concentrated by evaporation and re-suspended in water.

### 2.2. Surface Plasmon Resonance (SPR) Analysis of Florfenicol MIP and NIP Nanoparticles

The affinity of synthesized MIPs to FF was determined using SPR-2 biosensor system (Sierra Sensors GmbH, Hamburg, Germany) as described in the work of Altintas et al. (2015) [31]. Both MIP and NIP were covalently immobilized onto amine-derivatized gold chip via *N*-(3-dimethylaminopropyl)-*N*′-ethylcarbodiimide hydrochloride (EDC) activation. For this, an aliquot of either nanoparticle solution was diluted 1:10 with DI water and mixed with 5 mg/mL of EDC. This solution was then immediately injected onto the chip, on individual channels; up to 6000 response units were obtained from the bound nanoparticles. FF in water was then injected in both MIP and NIP-containing channels, at concentrations ranging from 0.001 to 1000 nM. The instrumental setup was: Flow rate: 30 μL/min, injection volume 80 μL, dissociation time: 150 s. Data was fitted to a 1:1 Langmuir binding model after subtraction of injection bulk effects using the Biaevaluation software. Kinetic data was analyzed using an SPR-2 analyzer.

### 2.3. Characterisation of NanoMIPs

Physical properties of the synthesized MIP nanoparticles were characterized using a Z-potential and hydrodynamic diameter (Z-average), and the quality of the production was evaluated using the poly-dispersity index (PDI). The MIP nanoparticles were measured at 25 °C using a Zetasizer Nano ZS-20 (Malvern instruments) operating at 4.0 mW and 633 nm with a fixed scattering angle of 173°. The samples were evaluated in 1 mL solutions contained inside capillary sample tubes of the nanoMips. Philips Tecnai 12 Bio Twin transmission electron microscope (TEM) was used to observe the morphology of the nanoparticles. One drop of nanoparticles dispersion was spread onto a coated copper grid, which was then dried at room temperature prior to TEM analysis.

### 2.4. Immobilization of MIPs onto the Surface of Microplate Wells and Assay Conditions

A homogenous solution of FF-MIPs nanoparticles in water (50 µg/mL) was prepared and mixed with polyvinyl alcohol (PVA) 0.2% (*w/v*). 50 µL of this solution was dispensed into each well of Nunclon^tm^ 96 well microplates (Thermo Fisher Scientific, Kamstrupvej, Denmark). The plate was left to dry completely overnight at room temperature and at 70 °C for 2 h, until a thin and homogeneous film coating formed in each well. Each well was washed three times with 200 µL of phosphate Buffered Saline Solution (PBS) buffer (pH 7.2); the wells were then blocked for 1 h with 200 µL of bovine serum albumin protein (BSA) 0.1% (*p/v*) and Tween solution 1.0% (*v/v*). After the blocking step, each well was washed three times with 200 µL of PBS buffer (pH 7.2), and then 100 µL of conjugated FF with horseradish peroxidase (AAT Bioquest^®^) in 20–300 ng/mL concentrations was dispensed into the wells, followed by incubation for 1 h. This was followed by washing with PBS (3 × 300 μL). Afterwards, 100 µL of HRP substrate mix solution in equal parts with Luminol enhancer^®®^ and stable peroxide solutions (Themoscientific, Rockford, IL, USA) was added to each of the test wells followed by incubation for 10 min. The plate was then read by determining the absorbance of each well at 450 nm using microplate reader DNM-9602 (Perlong, Beijing, China).

Binding competition between free FF (10–300 ng/mL) and FF-HRP was used to determine affinity of nanoMIPs immobilized onto wells.

To determine the specificity and selectivity of nanoMIPs for FF, four antibiotics were selected: oxytetracycline (OTC), flumequine (FMQ), thiamphenicol (TPH) and florfenicol amine (FFA). The assays were developed with nanoMIPs immobilized as above, but in this case, prior to the addition of HRP-FF, the different antibiotics were added.

Each experiment was conducted minimum three times, and each analysis was made in triplicate. The experimental data were analysed by the analysis of variance, and significant differences between means were measured using Tukey’s multiple range tests (Statgraphic version 4.0). A *p* level of 0.05 was used to determine significance.

### 2.5. FF Binding to NanoMIPs in Milk and Fish Samples

Milk and salmon samples were purchased in local supermarkets (Santiago, Chile) and were stored until needed. The salmon and milk were spiked with FF or HRP-FF (300 ng/g) for 3 h and homogenized at room temperature. Afterwards, 5 g or 5 mL of respective samples were taken from homogenized material and mixed with 10 mL of acetonitrile/dichloromethane (2:1). This suspension was blended for 10 min in multireax and centrifuged at 4500 RPM for 10 min at 4 °C. The supernatant was recollected and dried at 40 °C until a constant weight was obtained. The dry mass was resuspended in hexane/NaCl 10% a (*w/v*) aqueous solution and vortexed for 60 s, and the aqueous phase was recovered and filtered. The concentration of FF was determined in the supernatant using the assay described above. The blank sample was prepared similarly with the same procedure without spiking with FF.

### 2.6. Determination of MIP Shelf-Life

The microplates with immobilized nanoMIPs (see Section 2.5) were left for 6 weeks at 4 °C and at room temperature. The binding capacity and detection limit of FF in samples were measured using assay conditions described above.

## 3. Results and Discussion

### 3.1. Synthesis and Characterization of NanoMIPs

The method used to synthesise FF-MIP nanoparticles was based on using solid-phase approach developed by Piletsky and co-authors [31,32]. The physical properties of the FF-nanoMIPs depend on the manufacturing procedure. The integrity of binding sites in the polymers depends on the type and level of cross-linking which directly affects the morphology, binding selectivity, thermal stability and mechanical characteristics of the particles. Table 1 shows the results obtained from physicochemical characterization of MIPs performed by dynamic light scattering (DLS). The hydrodynamic diameter (Z-average) of FF-nanoMIPs was 123.1 ± 2.4 nm in stock. The dimensions (nm) of nanoMIPs are dependent on the type of material and preparation strategy with which they are manufactured. The size of FF-nanoMIPs reported here is consistent with other MIPs manufactured with materials and techniques similar to reported earlier, which were approximately 130 nm [31]. The size of nanoMIPs synthesized earlier by the co-precipitation method using poly(9-vinylcarbazole) (PVK) and poly(styrene-co-maleic anhydride) (PSMA) polymers showed significantly smaller sizes than those obtained here, between 70 and 80 nm as determined by DLS [33]. Additionally, when nanoMIPs were synthesized with polymers such as melamine and triethylamine by precipitation via centrifugation and washed with dichloromethane, sizes below 50 nm were obtained [34]. When the dispersion of FF-nanoMIPs underwent ultrasound treatment for 15, 30 and 60 min, no significant differences in size were observed (*p* > 0.05). These observed values as well as the values obtained from the polydispersity index (PDI) were all in the range of 0.41–0.44, which indicates stability of the colloidal system [35,36].

Additionally, the stability of the colloidal system was analyzed by measuring slipping planes values in the electrical layer of water/acetonitrile (Z-potential) which equals to −27.1 ± 0.3 mV. The FF-MIPs nanoparticles showed no significant difference (*p* > 0.05) in Z-potential and PDI before and after sonication, indicating a lack of coalescence or agglomerations between the particles.

**Figure 1:**
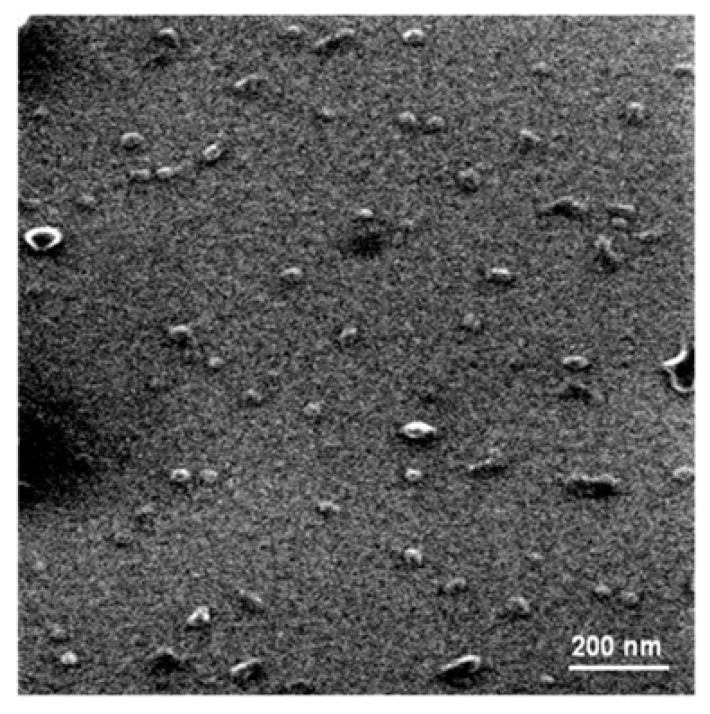
TEM micrograph NanoMIPs specific for florfenicol.

Figure 1 shows the TEM micrograph of FF-nanoMIP on a silica surface, wherein a defined regular oval-spherical structures were observed. The sizes mean of nanoMIPs determined by SEM was approximately 25 nm. On the other hand, the size (hydrodynamic diameter) determined by dynamic light scattering (DSL) was ~120 nm (shown in Table 1). The values determined using DSL (wet type analysis) were nearly 5-fold higher than those determined by SEM (dry type analysis), which indicates swelling of the polymers in water [37].

### 3.2. SPR Analysis of NanoMIPs Affinity

**Figure 2:**
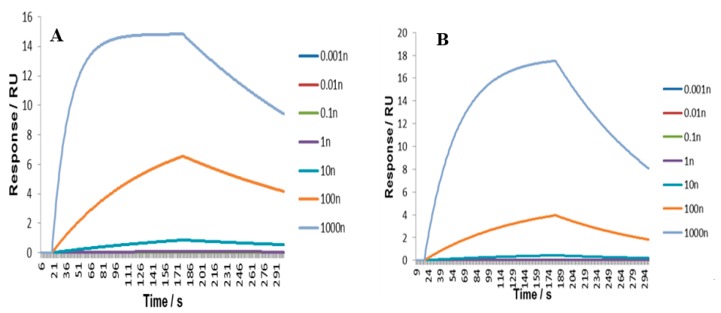
Surface Plasmon resonance (SPR) analysis of (**A**) florfenicol binding by florfenicol nanoMIP and (**B**) NIP.

To establish the selectivity of a MIP for a particular analyte, FF in this case, a non-imprinted polymer (NIP) is often synthesized in parallel with MIP [38]. A non-imprinted polymer (NIP) consists of the same constituents as the MIP except for the template molecule [39]. As the template molecule is responsible for the formation of the specific binding sites in MIP, a comparison of MIP and NIP made using the same composition would reveal the extent of polymer selectivity [40]. This difference is often referred to as an imprinting factor [41]. An SPR-based sensor was employed to study the affinity of nanoMIPs for FF. The respective binding curves for MIPs and NIPs are shown in Figure 2A,B. The affinity between nanoMIPs and antibiotic (FF) was investigated by calculating the dissociation constant (K_D_) according to the Langmuir binding model using the SPR-2 analyzer software [42]. This model is the most widely used method to calculate the total number of binding sites (N_t_) and the global mean affinity constant (K_0_) in homogeneous systems [43]. A K_D_ of 7.5 × 10^−8^ M was obtained for the MIP and 3.6 × 10^−7^ M for the NIP. Chi^2^ values were below 7 × 10^−4^, meaning there was a good fit between the theoretical binding model and the experimental data. The K_D_ value obtained in this work was similar to K_D_ values previously obtained for this type of nanoparticles [21,31]. Reported K_D_ for melamine, vancomycin and peptides were within 6.3 × 10^−8^–3.16 × 10^−9^ M. The nanoMIPs in practical terms resemble monoclonal antibodies. There is a different binding behavior of nanoparticles, with FF-MIP reaching saturation earlier (66–171 s) than NIPs (99–178 s) and with a slower dissociation, as shown in the sensor grams. The K_D_ and adsorption capacity (Q_MAX_) for MIPs were described using a model with two kinds of binding sites, low affinity sites and high affinity sites. It is suggested that a dual-site Langmuir binding model might better describe template binding by MIPs [44].

### 3.3. Binding of FF to Immobilized NanoMIPs in ELISA-Type Assay

**Figure 3:**
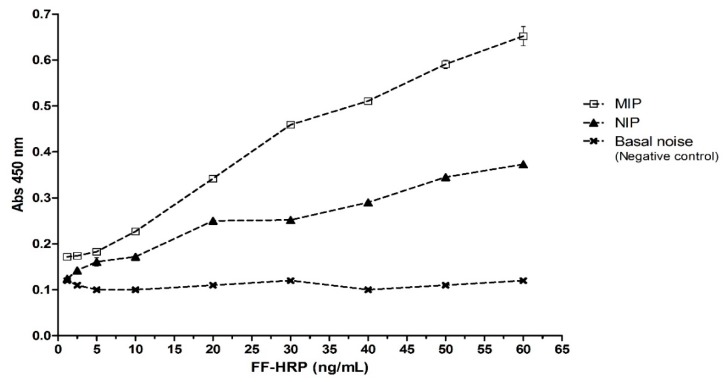
Detection of FF on ELISA microplates with immobilization of nanoMIPs.

The immobilization of the nanoMIPs in microplate wells was based on the modified protocol published earlier [45]. A simple immobilization procedure was designed for deposition of stable coatings of FF-nanoMIPs on the surface of microplate wells. A total of 40 µL (2.8 µg particles per well) were loaded into each well with stock solution of FF-nanoMIP, followed by total evaporation of the solvent. The nanoMIPs were immobilized through physical adsorption to the walls of polystyrene microplates by hydrophobic binding. To develop the assay, coated wells were incubated with FF conjugate with horseradish peroxidase (EC 1.11.1.7) (FF-HRP). HRP is commonly used in ELISA for efficient colorimetric detection [29]. The incubation was followed by washing with PBS buffer and color development through a reaction with luminol enhancer^®®^. Uncoated wells were similarly treated as a control (basal noise). Results shown in Figure 3 indicate that signal intensity, measured at 450 nm, is directly proportional to the concentration of HRP-FF added to MIP-coated wells, indicating an adequate binding between antibiotic and MIP. Significant differences were observed with test concentrations between 10 and 60 ng/mL, but when concentrations were lower, in the range of 1.2–5 ng/mL, no significant differences were observed. The results show that much higher binding of FF-HRP was seen in the case of the nanoMIPs compared with either nanoNIPs or bare wells. The responses obtained for FF-nanoNIPs incubated with HRP-FF in all cases were smaller than the responses observed for MIPs (generally less than 50% intensity). These results prove a specific interaction between the analyte portion of FF−HRP and the MIPs. The background noise and possible non-specific binding of the labeled antibiotic with the empty wells was less than 0.108. Moreover, this test also proves that MIPs remain attached to the microplate well surfaces, even after several washes with PBS. It was possible to conclude that stable coatings could be achieved by allowing a solution of nanoMIPs to evaporate to dryness within each of the microplate wells. With this experimental strategy, it was possible to establish a stable and efficient method to measure FF in solution.

### 3.4. Competitive Binding of FF and HRP-FF in ELISA-Like Assay

**Figure 4:**
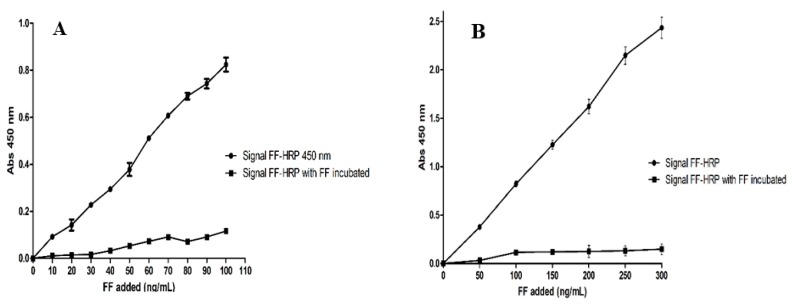
Competition between HRP-FF and free FF for binding to immobilized FF-nanoMIPs (**A**) 10–100 ng/mL concentrations and (**B**) 50–300 ng/mL concentration of FF.

Figure 4A,B show competitive binding of FF and FF-HRP (used in the concentration ratio 1:1). Using equal amounts of FF and HRP-FF at 50–300 ng/mL concentrations (Figure 4A) and 10–100 ng/mL concentrations (Figure 4B) allowed us to assess direct competition of the free and labeled target at equilibrium conditions. It was observed that FF (unlabeled) is able to quench the signal emitted by HRP-FF probe at all concentrations evaluated (10 to 100 ng/mL, Figure 4A and 50 to 300 ng/mL, Figure 4B). This result is attributed to the displacement and specific binding of FF to the nanoMIP. When low concentrations were tested (10 to 30 ng/mL, Figure 4A), the sensitivity of method decreased. The signals detected in these concentrations were similar to those detected in the baseline noise of the test shown in Figure 3.

### 3.5. Cross-Reactivity of NanoMIPs

**Figure 5:**
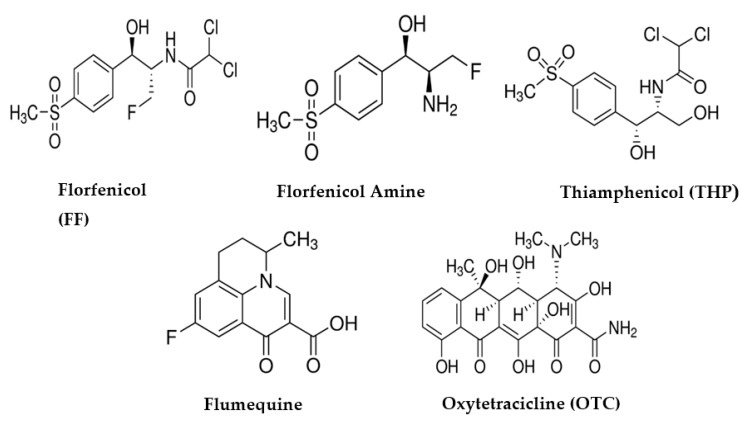
Chemical structures of antibiotics used in this work.

Determination of specificity and cross-reactivity of nanoMIPs immobilized in the microplate are crucial for diagnostics [46]. A competition assay was performed against four types of antibiotics (AB): two with totally different structures to FF, such as (1) oxytetracycline (OTC) and (2) flumequine (FMQ); and two with very similar structures to FF, such as (3) thiamphenicol (TPH) and (4) florfenicol amine hydrochloride (FFA), a FF intermediate from animal primary metabolism [47]. Their chemical structures are illustrated in Figure 5. Antibiotics studied in this work were added to assay in the concentrations 1:1 in relation to FF-HRP (10–300 ng/mL).

**Figure 6:**
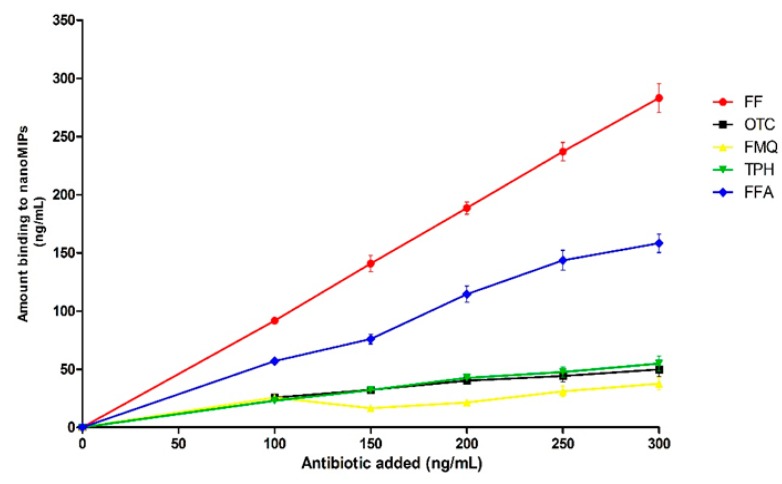
Competition between HRP-FF and antibiotics structurally similar and different to FF for binding to immobilized FF-nanoMIPs.

The cross-reactivity of antibiotics used at 10–100 ng/mL was assessed by measuring decrease of signal produced by HRP-FF added at the same concentration. The results shown in Figure 6 indicate very low binding of all non-related targets to nanoMIPs. Binding of FFA to MIP is the highest observed in this work as compared with other antibiotics, which was attributed to a very close structural similarity between FF and FFA.

### 3.6. HRP-FF Detection in Food Matrices

In this work, we analyzed two food matrix types; salmon muscle (fillet) and fluid milk (31 g/L of fat) in order to determine the feasibility of recovering the antibiotic from the samples. This can be measured by the immobilized FF-nanoMIPs; as the first experimental strategy, the samples (milk and salmon) were spiked with FF-HRP at 20 to 300 ng/mL concentrations. After the incubation process, analyte was recovered and compared with positive control, where the same concentrations of FF-HRP were added directly to wells with immobilized MIPs.

The results for the milk sample are shown in Table 2 and demonstrate a good recovery performance. Among the highest concentrations tested, there was a recovery of 90%. Between concentrations of 80 and 60 ng/mL that were spiked, a 95% recovery was obtained. A significant decrease in the analyte recovery at the lowest concentrations tested was observed; it was only possible to recover 80% of the analyte spiked. For the salmon fillet sample, the results are shown in Table 3. A lower recovery yield was obtained compared to the milk sample; this result can be attributed to a large number of interferences present in this type of sample of greater complexity of its components. The recovery rate among the highest concentrations (150, 200, 250 and 300 ng/mL) only reached 71.9 ± 3.44% to 77.4 ± 2.68%. The best recovery levels were observed in the concentrations of 90 and 100 ng/mL where the percentages 84.5 ± 3.66% and 87.4 ± 1.44% were found, respectively. For the lowest concentrations tested, the values could not be detected. The decreased recovery could be attributed to interferences in the food matrix or the ion suppression effect. The ion suppression effect, where precision and accuracy decrease with respect to control, increase the relative error [48]. This phenomenon causes a reduction of signal strength and performance and, as a consequence, affects the analytical method with respect to precision, accuracy and sensitivity [49]. Another factor that may be attributable to the results obtained corresponds to solvents used in the extraction process. This parameter has been described as key in the recovery percentage and therefore directly affects the accuracy of the method [14]. Furthermore, Zou et al. (2013) [50] reported that the signal suppression of FF was observed in casings and that the signal reinforcement was observed in swine muscle.

The matrix effects may arise from the co-extraction or co-eluting matrix components during the sample pretreatment. When sample pretreatment with good selectivity was used, the matrix effects could be reduced significantly and the producibility and accuracy of the results was also improved [14]. Additionally, both food matrices possessed a considerable amount of interfering molecules such as histidine, methionine, glycine, phenylalanine, arginine as well as fructose.

### 3.7. Detection of FF in Food Matrices

The presence of spiked FF in food samples was determined by measuring a decrease in HRP-FF signals caused by competition of free and labeled FF over binding to nanoMIPs binding sites. The food samples were spiked with FF (300 ng/mL or g, depending on milk or salmon), and following procedure described earlier, FF was extracted and analyzed. The matrix effect was minimized using solvents which allow avoiding having proteins and sugars in the extraction solution. No significant interference with matrix components was detected in the signal emitted by FF-HRP. Signal from FF was assessed for 100 μL of extracts and the respective dilutions (1/10 and 1/100), tested in triplicate on three plates independently. With the goal to assess shelf-life of nanoMIPs coated microplates, the assay was performed in fresh plates (0 week) and plates stored for six weeks. The plates were stored at room temperature (RT1) and at 5 °C (RT2). The results shown in Table 4 indicate good signal recovery for all FF concentrations in spiked milk samples. The highest levels of recovery (%) in milk samples observed in the first storage weeks (0 and 1) in RT1 condition were 85.84 ± 5.41% and 85.12 ± 4.22%, equivalent to 257.52 ± 4.32 and 255.36 ± 3.18 ng/mL, respectively. During the same period under RT2 conditions, the recovery values were 80.71 ± 3.22% and 80.82 ± 3.54%, equivalent to 242.13 ± 2.62 and 242.46 ± 2.32 ng/mL, respectively. At both conditions (RT1 and RT2), after 6 weeks of storage the recovery percentage decreased to 80.34 ± 4.76% (241.02 ± 4.76 ng/mL) in RT1 and 78.34 ± 6.13% (235.02 ± 4.76 ng/mL) in RT2. In salmon samples, the recovery rate at both storage conditions was slightly lower than that observed for milk samples. At RT1 condition the recovery percentage from salmon samples was 71.48 ± 4.12% (214.45 ± 2.12 ng/mL) after six weeks of storage recovering; for the same period, at the RT2 condition, the recovery percentage was 74.3 ± 0.65% (224.45 ± 1.12 ng/mL). This assay shows that the use of immobilized nanoMIPs allows the recovery of approximately 80% of the FF antibiotic after six weeks of storage under different temperature conditions of the device and two different kinds of samples. No appreciable changes in response were detected for microplates stored for six weeks. The good stability of MIPs stored at room temperature is very important for avoiding problems associated with the need for cold chain supply.

It has been successfully demonstrated in previous research that nanoMIPs can be used to detect antibiotic such as gestamicin with performance compared to the ELISA method [51]. The recovery (ng) of FF in milk and fish extracts, showing the storage effect and shelf life of nanoMIP immobilized in microplates, was demonstrated through the new method. This optimized method achieves sufficient sensitivity to detect the presence of FF compared to competitive technologies such as the ELISA method. ELISA is one of the most used diagnostic tests and it is based on the use of antibodies to quantify the molecule of interest. The main advantage of nanoMIPs in the diagnosis is the possibility of direct antibody replacement in standard ELISA assays with minimal modification of the immobilization and assay protocol. The nanoMIP, thanks to its stability, profitability and easy production, is a promising alternative to antibodies in assays and sensors.

## 4. Conclusions

This study demonstrates that nanoparticles imprinted with FF have a uniform size and good physicochemical stability, as determined by Z-potential and a low polydispersity index. Their binding to FF is characterized by K_D_ of 7.5 × 10^−8^ M. The nanoMIPs were successfully immobilized in wells of ELISA plates for the construction of a prototype of assay for detection of antibiotics. With this experimental strategy, it was possible to establish a stable and efficient method to detect antibiotics by using colorimetry. The FF can be detected, potentially, using this assay at concentrations of 10–100 ng/mL. The cross-reactivity studies with the other antibiotics indicated a high selectivity of MIPs. The nanoMIP-coated microplates do not require low temperatures for storage. FF can be detected in extracts of milk and fish. It is possible to use the proposed approach to design a MIP-based ELISA-like assay for the detection of FF.

## Figures and Tables

**Table 1:** Physical properties of nanoMIPs suspension with and without sonication. Different letters (a,b,c) indicate significant differences (*p* < 0.05).

Sonicated Time (min)	Z-Average (nm)	PDI	Z-Potential (mV)
0	123.1 ± 2.4 ^a^	0.42 ± 0.0 ^a^	−27.1 ± 0.3 ^a^
15	124.2 ± 3.3 ^a^	0.41 ± 0.0 ^b^	−28.4 ± 0.9 ^b^
30	123.9 ± 1.9 ^a^	0.44 ± 0.1 ^c^	−28.3 ± 0.9 ^b^
60	123.4 ± 2.6 ^a^	0.42 ± 0.0 ^a^	−27.4 ± 1.9 ^a^

**Table 2:** Testing of milk sample spiked with FF-HRP.

Spiked (ng/mL)	Measured Concentration (ng/mL)	Recovery %
300	269.64 ± 9.77	89.88 ± 3.99
250	237.91 ± 5.48	95.16 ± 2.68
200	179.47 ± 2.26	89.74 ± 1.39
150	135.81 ± 1.32	90.54 ± 1.08
100	91.23 ± 2.72	91.23 ± 3.33
90	82.29 ± 1.81	91.43 ± 2.47
80	76.38 ± 1.22	95.48 ± 1.87
70	67.15 ± 0.46	95.92 ± 0.81
60	56.55 ± 0.63	94.24 ± 1.29
50	41.77 ± 2.57	83.54 ± 3.31
40	32.46 ± 0.58	81.16 ± 1.78
30	25.04 ± 1.04	83.46 ± 2.28
20	15.62 ± 2.15	78.10 ± 3.18

**Table 3:** Testing of salmon fillet sample spiked with FF-HRP.

Spiked (ng/mL)	Measured Concentration (ng/mL)	Recovery %
300	232.22 ± 8.03	77.4 ± 2.68
250	193.95 ± 5.22	77.6 ± 2.09
200	154.36 ± 6.77	77.2 ± 3.38
150	107.85 ± 5.16	71.9 ± 3.44
100	87.43 ± 1.44	87.4 ± 1.44
90	76.09 ± 3.29	84.5 ± 3.66
80	30.65 ± 3.27	38.3 ± 4.69
70	24.45 ± 1.18	34.9 ± 1.09
60	16.76 ± 2.43	27.9 ± 2.08
50	6.94 ± 0.57	13.9 ± 1.14
40	undetermined	No recovery
30	undetermined	No recovery
20	undetermined	No recovery

**Table 4:** Recovery (ng) of FF spiked at 300 ng concentrations in milk and fish extracts showing storage effect and shelf life of nanoMIPs immobilized on microplates.

	Storage Condition			
	Room Temperature (RT1)		Refrigerated Temperature (RT2)	
	Milk	Salmon	Milk	Salmon
Week	Recovery (%)			
0	85.84 ± 5.41	80.39 ± 4.28	80.71 ± 3.22	79.82 ± 6.22
1	85.12 ± 4.22	78.59 ± 3.54	80.82 ± 3.54	79.66 ± 3.65
2	83.37 ± 3.23	77.18 ± 4.65	80.71 ± 4.35	78.85 ± 3.34
3	84.03 ± 2.36	75.48 ± 4.12	80.41 ± 5.18	78.81 ± 4.12
4	83.28 ± 4.22	73.85 ± 4.22	79.80 ± 4.65	77.18 ± 3.23
5	81.94 ± 3.23	72.22 ± 6.11	78.55 ± 5.76	75.55 ± 4.65
6	80.34 ± 4.76	71.48 ± 4.12	78.34 ± 6.13	74.82 ± 3.6
